# Supplementary material for: Nucleosome positioning stability is a modulator of germline mutation rate variation across the human genome
Source: Nat Commun. 2020 Mar 13;11:1363. doi: 10.1038/s41467-020-15185-0 (PMC7070026; doi:10.1038/s41467-020-15185-0)
Supplement: Supplementary file 1 — Supplementary Information [file 41467_2020_15185_MOESM1_ESM.pdf]

Supplementary Information for

Li et al. “Nucleosome positioning stability is a modulator of germline mutation rate variation across the human genome”

Supplementary Data and Figures

Supplementary Data 1 Coefficients of variables and other information from the full regression models for different mutation types (in a separate Excel file). Note that for each of the categorical variables, the first category was used by the regression model as reference category (other categories were compared with the reference category) and thus there is no coefficient for that category. The statistics (4th column) and p-values (5th column) in the table were from Wald tests defaultly produced by 'bayesglm' (shown for reference), which are different from the likelihood ratio test-based p-values and were not used in our discussion.

Supplementary Data 2 Results of likelihood ratio tests (LRT) and the McFadden's pseudo R^2 of full and reduced models (in a separate Excel file).

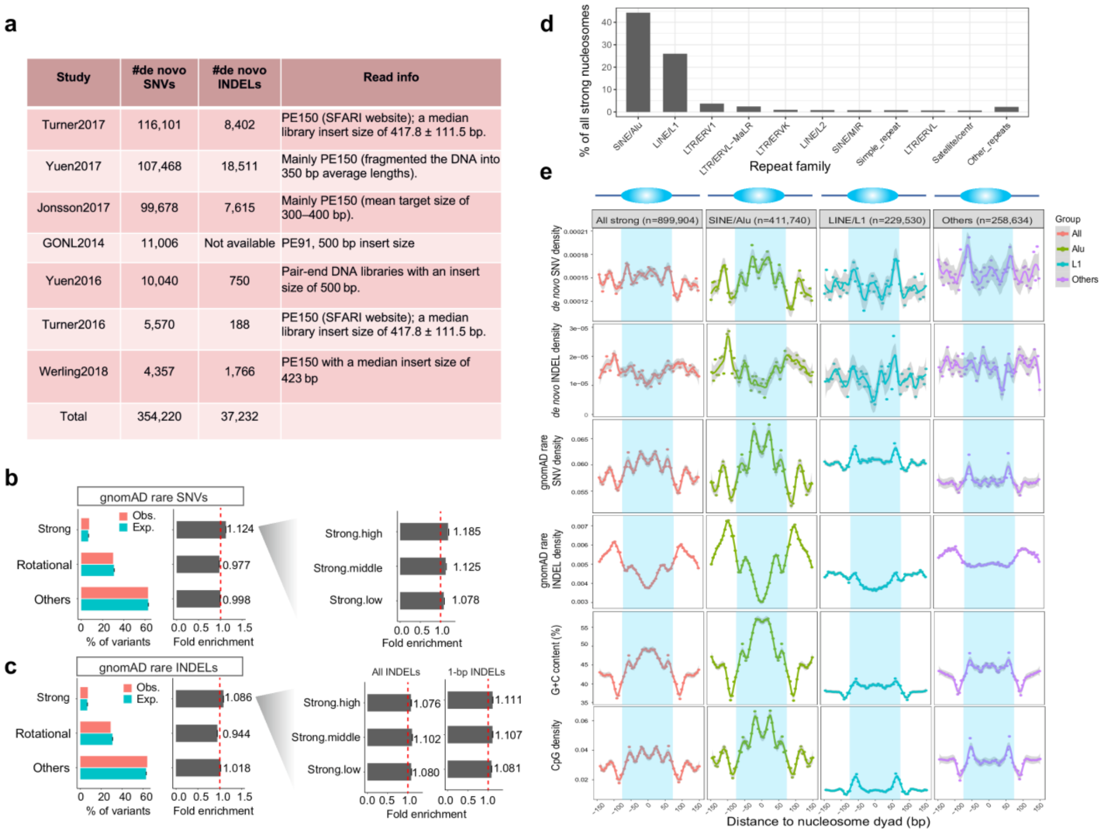

Study	#de novo SNVs	#de novo INDELs	Read info
Turner2017	116,101	8,402	PE150 (SFARI website); a median library insert size of 417.8 ± 111.5 bp.
Yuen2017	107,468	18,511	Mainly PE150 (fragmented the DNA into 350 bp average lengths).
Jonsson2017	99,678	7,615	Mainly PE150 (mean target size of 300–400 bp).
GONL2014	11,006	Not available	PE91, 500 bp insert size
Yuen2016	10,040	750	Pair-end DNA libraries with an insert size of 500 bp.
Turner2016	5,570	188	PE150 (SFARI website); a median library insert size of 417.8 ± 111.5 bp.
Werling2018	4,357	1,766	PE150 with a median insert size of 423 bp
Total	354,220	37,232	

Supplementary Figure 1 Mutations in different nucleosome contexts. (**a**) Information of the *de novo* mutation datasets from seven studies used in analysis. (**b**) Fold enrichment/depletion of gnomAD extremely rare SNVs in different nucleosome contexts. ‘Strong’, translationally stable positioning; ‘Rotational’, rotationally but not translationally stable positioning; ‘Others’, the remaining genomic regions. On the left is the fold enrichment for three subgroups of strong nucleosomes with different stabilities. Error bars depict 95% confidence intervals. (**c**) Fold enrichment/depletion of gnomAD INDELs in different nucleosome contexts. When using all INDELs the ‘strong.high’ group does not have a higher mutation rate than other two groups, but if using the 1-bp INDELs ‘strong.high’ does have the highest mutation rate among the three groups. We speculated that there may be more false negatives of longer INDELs in the ‘strong.high’ group. (**d**) Top 10 repeat families that are associated with strong nucleosomes. (**e**) Meta-profiles of SNV/INDEL densities (*de novo* or extremely rare variants) around all strong nucleosomes, or in different repeat-associated subgroups. At the bottom are the G+C content and CpG content profiles. Source data are provided as a Source Data file.

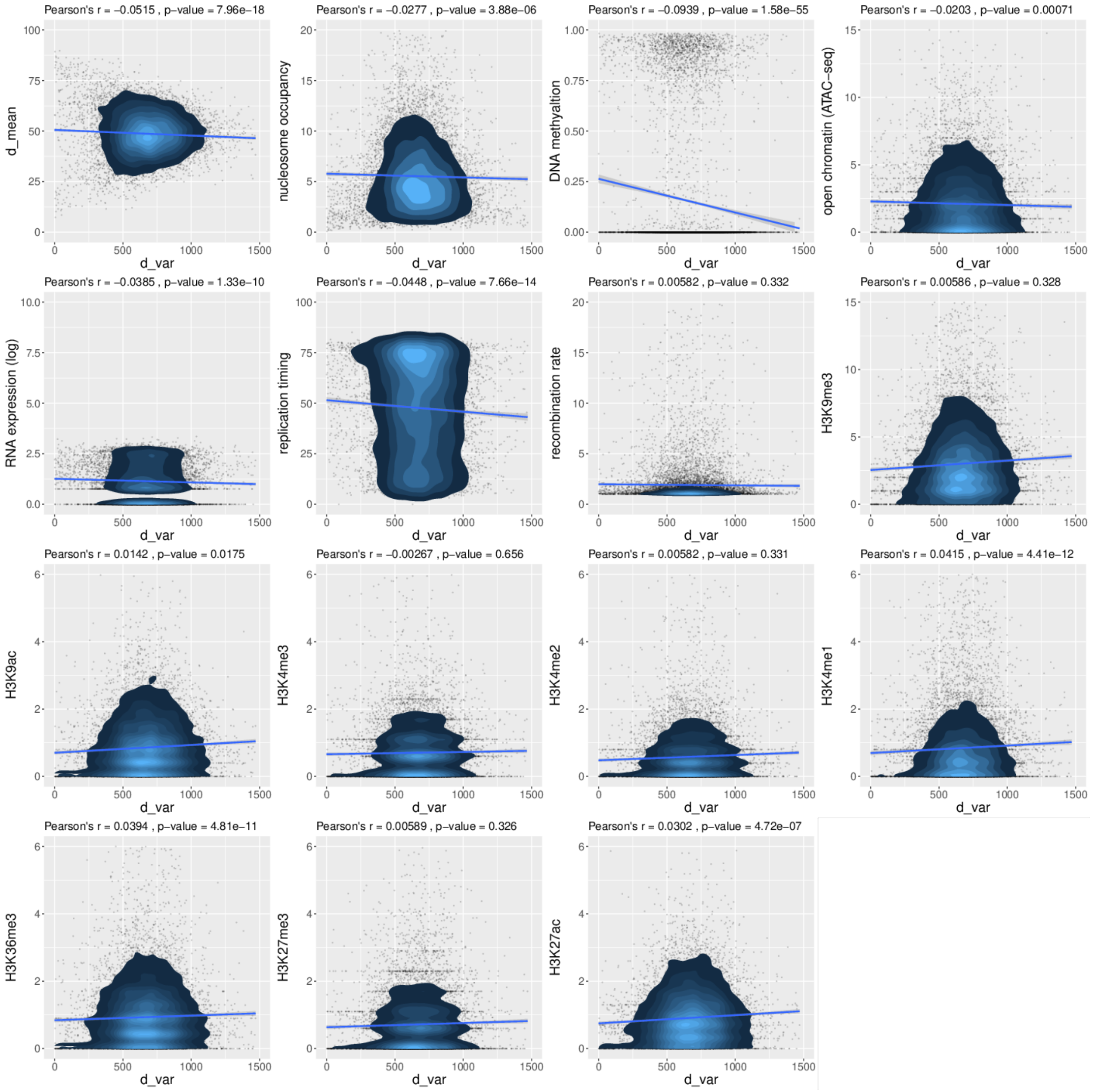

Supplementary Figure 2 Correlation analysis between nucleosome positioning stability (*d_{var}*) and other factors. On the top of each panel are the Pearson's correlation coefficients and the corresponding p-values. We randomly chose 1% (27,847 sites) of genomic sites used in logistic models for this analysis. Source data are provided as a Source Data file.

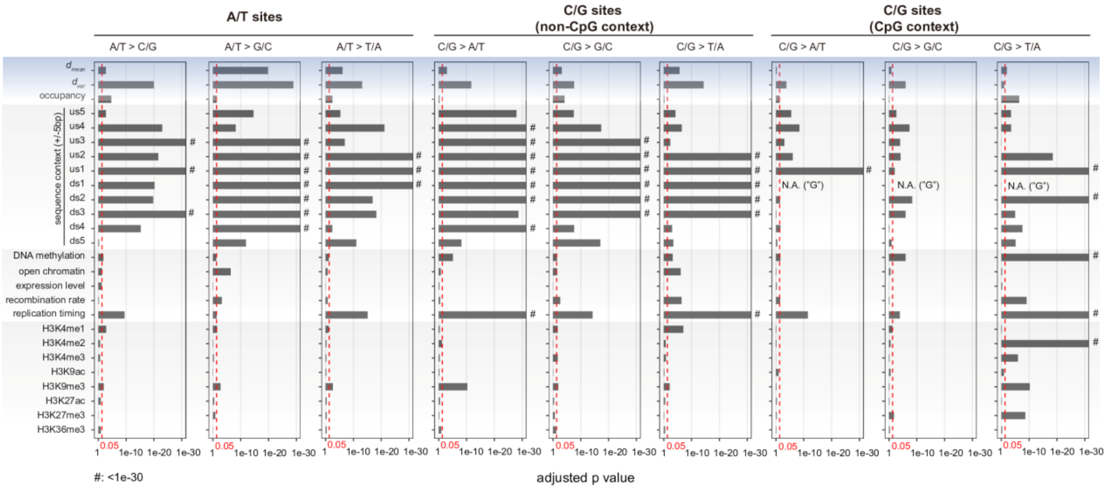

Supplementary Figure 3 Results of statistical tests for nine individual SNV mutation types. C/G sites in non-CpG contexts and C/G sites in CpG contexts were tested separately. The red vertical lines represent the significance cut-off (0.05) for the adjusted p values (Benjamini–Hochberg correction). ‘us’, upstream; ‘ds’, downstream. ‘#’ means adjusted p < 1e-30. Source data are provided as a Source Data file.

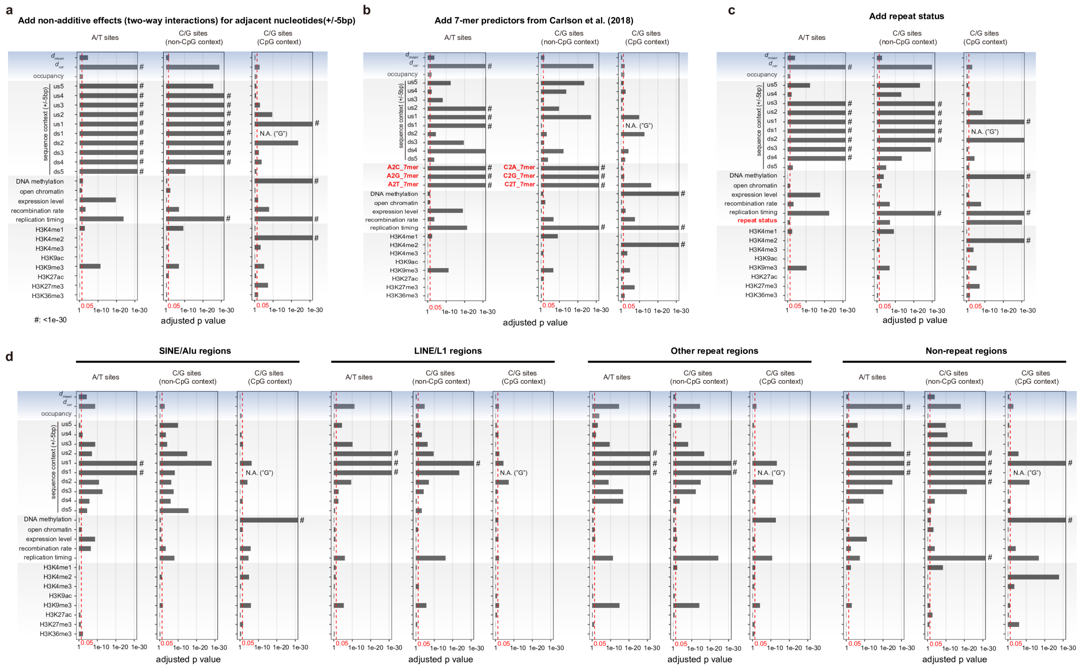

Supplementary Figure 4 Results of statistical tests when considering two-way interactions of adjacent nucleotides, 7-mer mutability estimates from Carlson et al. and repeat status. (**a**) Adding the two-way interactions for ±5 nucleotides in the regression models. (**b**) Adding the 7-mer mutability estimates from Carlson et al. as predictors in the regression models. (**c**) Adding repeat status as a predictor in the regression models. (**d**) Running regression models for regions associated with different repeat contexts separately. We tested SNVs at A/T sites, C/G sites in non-CpG context and C/G sites in CpG context separately. The red vertical lines represent the significance cut-off (0.05) for the adjusted p values (Benjamini–Hochberg correction). 'us', upstream; 'ds', downstream. '#' means adjusted p < 1e-30. Source data are provided as a Source Data file.

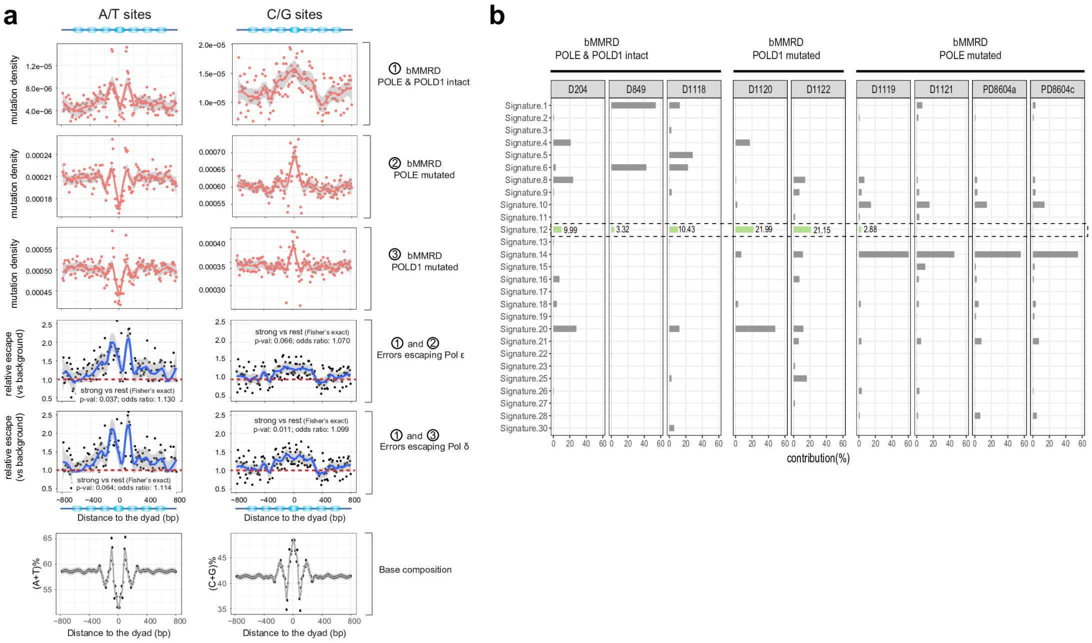

Supplementary Figure 5 Analysis of related mutational processes using bMMRD data. (**a**) Mutation profiles around strong nucleosomes for bMMRD cancer genomes and the estimated relative escape ratios of Pol ε or Pol δ, for mutations at A/T sites and C/G sites respectively. Fisher's exact test was used for testing the association of strong-nuclesome regions (dyad±95bp) with differential polymerase performance. (**b**) Comparison of the contribution of COSMIC mutational signatures predicted by MutationalPatterns in different bMMRD genomes. Highlighted is Signature 12, which shows a particularly high contribution in POLD1-muated bMMRD samples.

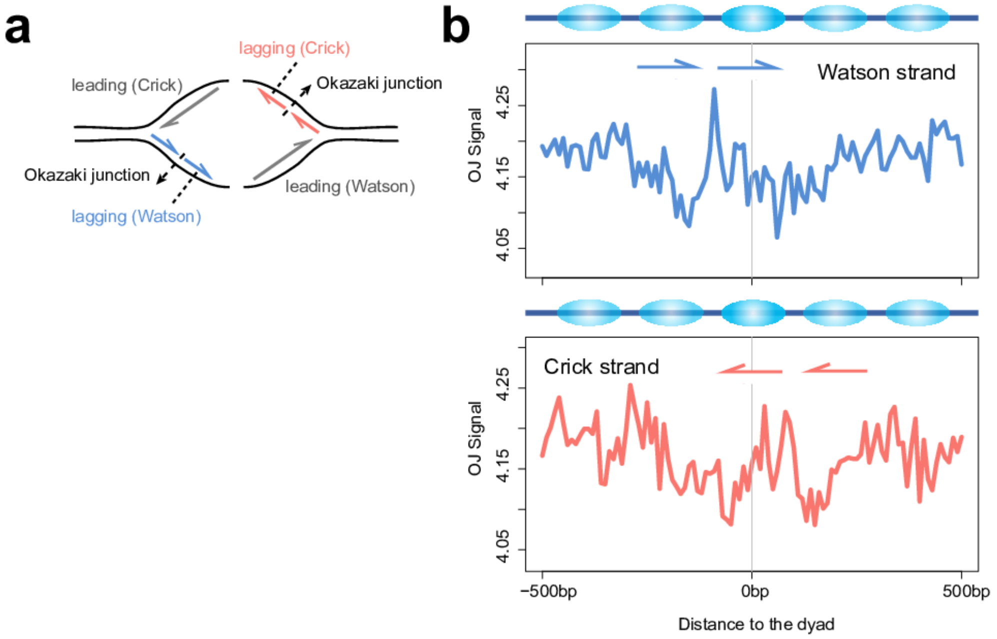

Supplementary Figure 6 Analysis with OK-seq data. (**a**) Schematic illustrating replication strands and Okazaki junctions (OJs). (**b**) Meta-profile of the density of Okazaki junctions inferred from alignments of OK-seq reads around strong nucleosomes (high-mappability). OJ signals for Watson strand and Crick strand were plotted separately. Replication directions of Okazaki fragments are shown by arrows. Source data are provided as a Source Data file.

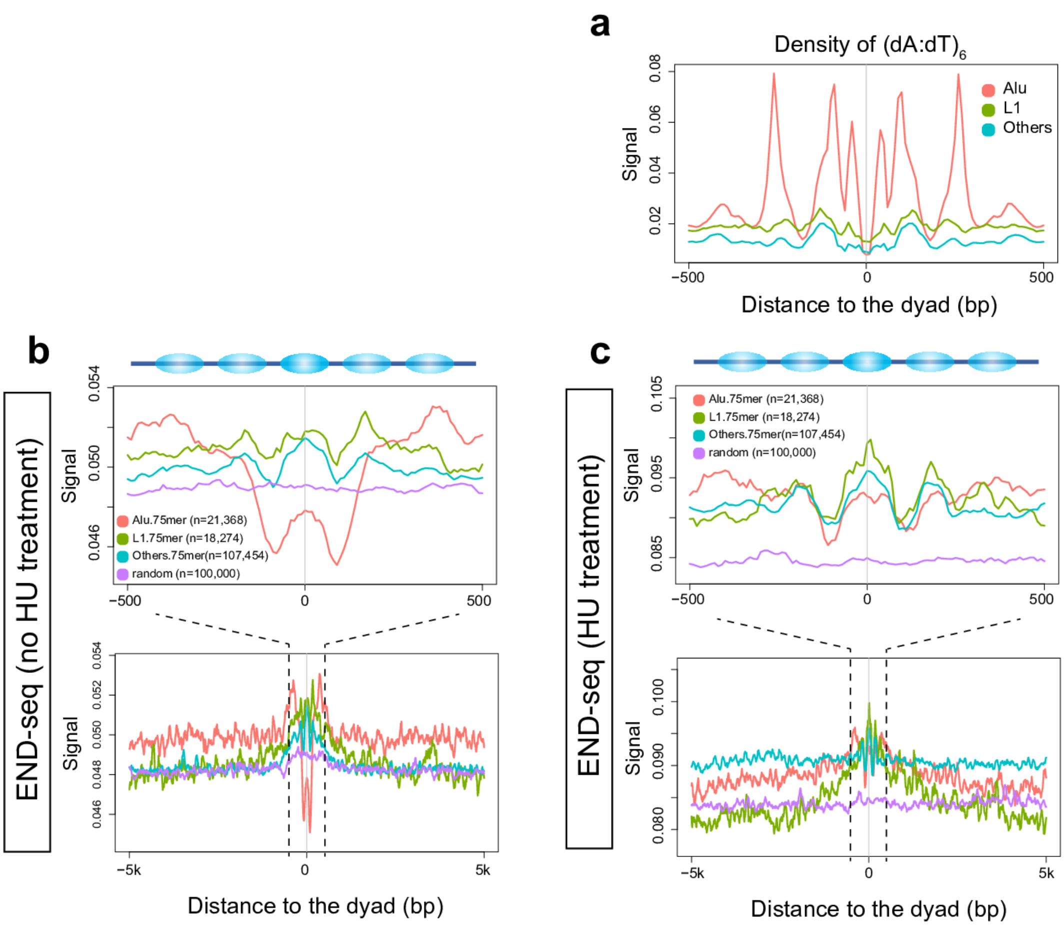

Supplementary Figure 7 Analysis related to the DSBs around strong nucleosomes. (**a**) Density of poly(dA:dT) tracts (based on occurrence of $(dA:dT)_6$ motifs) around strong nucleosomes. (**b-c**) Signal of DSBs based on the END-seq data around strong nucleosomes associated with different repeat elements. Only the strong nucleosomes of high 75-mer mappability within ±500bp were considered. Numbers of usable strong nucleosomes for each group are given in the brackets. HU (hydroxyurea) is a replicative stress-inducing agent. Source data are provided as a Source Data file.

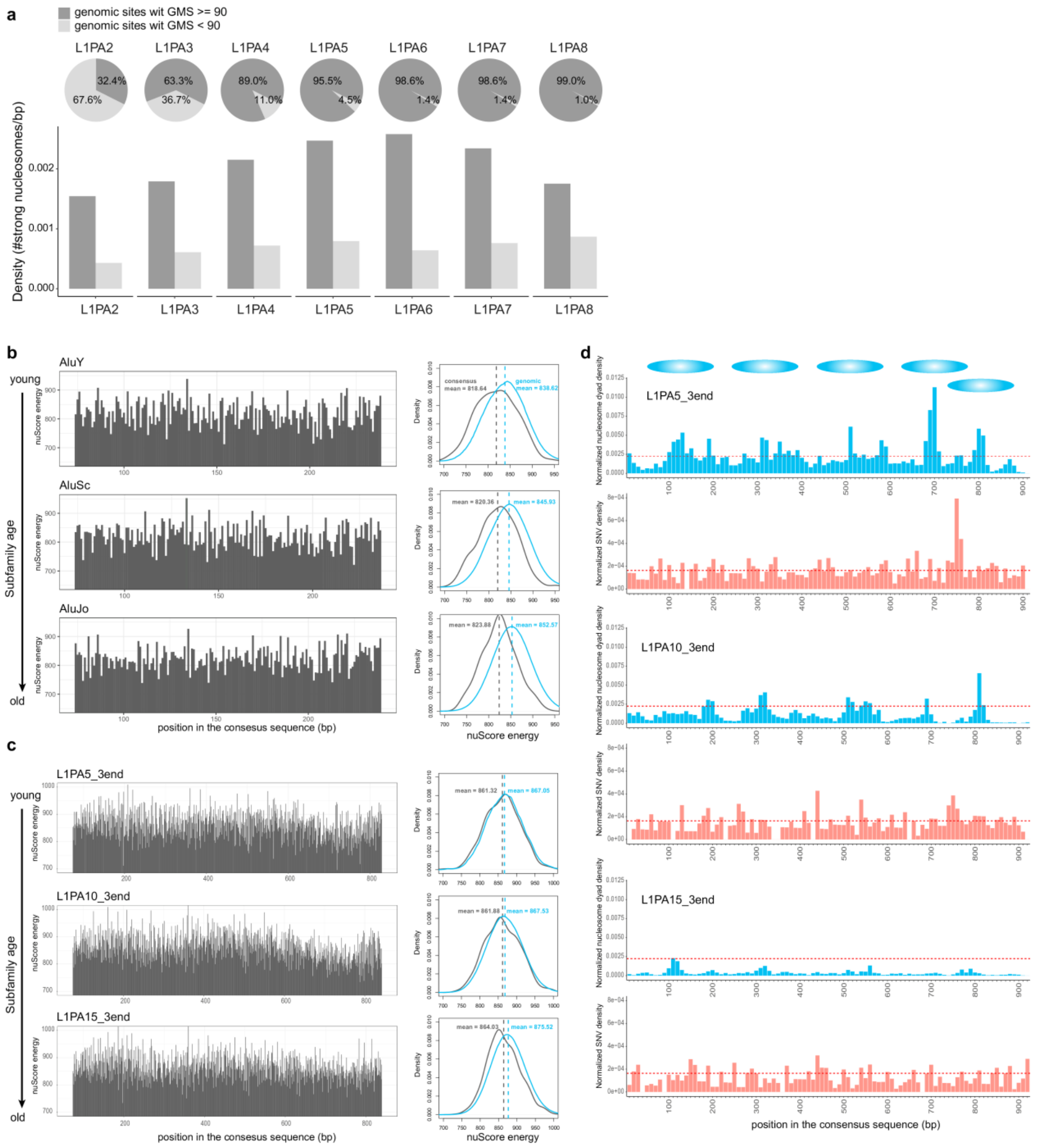

Supplementary Figure 8 Additional analysis about repeat subfamily ages and strong nucleosomes. (**a**) At the top are the fractions of each young L1 subfamily with different mappabilities (GMS >= 90 or GMS < 90). At the bottom are the densities of strong nucleosomes for regions with different mappabilities in each subfamily. (**b**) nuScore-estimated per-base nucleosome deformation energies along three Alu subfamily consensus sequences. On the right are the comparisons of deformation energy distributions of the consensus sequences (ancestral states) and those of current genomic regions for the three subfamilies respectively. The deformation energy profiles of the consensus sequences are similar, but the average deformation energies increase over time, with older Alu subfamilies displaying larger differences relative to the consensus. (**c**) Similar to (b), but for three example L1 subfamilies. (**d**) Barplots for normalized densities of strong nucleosome dyads and *de novo* SNVs along the consensus sequences of three L1 subfamilies, using 10-bp bins. Several loci that are enriched for dyads of strong nucleosomes are shown on the top with ellipses. The red dash lines represent the average densities for the L1PA5 subfamily. The densities of strong nucleosome dyads and *de novo* SNVs appear to decrease over evolutionary time. Source data are provided as a Source Data file.
