# Supplementary material for: Nucleosome positioning stability is a modulator of germline mutation rate variation across the human genome
Source: Nat Commun. 2020 Mar 13;11:1363. doi: 10.1038/s41467-020-15185-0 (PMC7070026; doi:10.1038/s41467-020-15185-0)
Supplement: Supplementary file 3 — Description of Additional Supplementary Files [file 41467_2020_15185_MOESM3_ESM.pdf]

# Description of Additional Supplementary Files

File Name: Supplementary Data 1
Description: Coefficients of variables and other information from the full regression models for different mutation types.

File Name: Supplementary Data 2
Description: Results of likelihood ratio tests (LRT) and the McFadden's pseudo R2 of full and reduced models.
